# Territorial competition and the evolutionary loss of sexual size dimorphism

**DOI:** 10.1007/s00265-014-1870-0

**Published:** 2015-01-17

**Authors:** Ulrike Odreitz, Kristina M. Sefc

**Affiliations:** Institute of Zoology, University of Graz, Universitätsplatz 2, 8010 Graz, Austria

**Keywords:** Contest competition, Territoriality, Body size, Resource-holding potential, Cichlidae

## Abstract

Non-sexual social selection can underlie the evolution of sexually monomorphic phenotypes. A causal relationship between territorial competition and sexual monomorphism predicts that male and female competitors should employ similar contest behavior and that contest outcome should depend on the same traits in males and females. We test this prediction in a sexually monomorphic cichlid fish of the genus *Tropheus,* in which males and females defend individual feeding territories. Lineages basal to *Tropheus* are sexually dimorphic and have non-territorial females, suggesting that a switch to female territoriality and loss of sexual dimorphism occurred in the *Tropheus* lineage. We compare rates of agonistic behavior and the effects of body size asymmetries on competitive success between male-male and female-female contests in an experimental setup. Body size asymmetry had the same effect in male and female contests, being negatively correlated with contest duration and positively correlated with the probability of winning. Male and female winners employed the same rates of frontal and lateral displays as well as charges against their opponents. Contest duration was longer in females. In tied contests, females displayed more than males. Our data suggest that intraspecific contest competition for territories selects for large body size in both sexes and support a link between the evolution of female territoriality and the loss of sexual size dimorphism in *Tropheus*.

**Electronic supplementary material:**

The online version of this article (doi:10.1007/s00265-014-1870-0) contains supplementary material, which is available to authorized users.

## Introduction

Ornaments, weapons, and large body size are associated with competitive and mating success in many animal species. Competition for reproductive opportunities is frequently more severe in one sex than in the other, which explains the widespread occurrence of sexual dimorphism in sexually selected traits. In contrast, competition over non-sexual, e.g., ecological, resources is more likely to be balanced between sexes and may favor the display of elaborate traits associated with competitive success in both males and females (West-Eberhard 1983; Amundsen 2000; Kraaijeveld et al. 2007; Tobias et al. 2012). Consistent with predictions regarding the effect of (non-sexual) social selection on phenotype evolution (West-Eberhard 1983), similarity in the social roles of males and females, as it is seen in species with male and female territoriality, was found to coincide with sexual monomorphism in body size and signal traits (Wolf 1975; Whittingham et al. 1992; Tobias et al. 2011).

The link between female territoriality and sexually monomorphic phenotypes can take different shapes. Monomorphism in a trait not only can result from competition-induced selection on that trait in both sexes but can also facilitate female territoriality after having evolved in response to a different selection pressure (Kraaijeveld et al. 2007). Consider scenarios of the evolution of a sexually monomorphic species with male and female territoriality from a sexually dimorphic ancestor with non-territorial females. Female territoriality can arise, for example, in the wake of a change in trophic resource use. The concept of economic resource defendability (Brown 1964) predicts that animals will defend feeding territories when food is distributed at moderate densities, is spatially and temporally predictable, and is renewable (Grant 1993). Correlations between resource use and territoriality have indeed been observed in different taxa (Brown 1964; Ostfeld 1990; Roberts and Ormond 1992; Grant 1997). Following a shift to territoriality, intraspecific competition for territories will expose females to selection on traits associated with resource-holding potential. If the traits influencing competitive success are the same in males and females (e.g., Batista et al. 2012), selection arising from competition will work against sexual dimorphism in these traits (Rubenstein and Lovette 2009). In contrast, females and males may rely on different traits and different behavioral strategies for competitive success (e.g., Draud et al. 2004; Arnott and Elwood 2009; Milner et al. 2010), and territorial competition would not select for the same traits in males and females. In this case, the loss of sexual dimorphism would have to occur for reasons other than territoriality.

Alternatively, if sexual monomorphism evolves prior to female territoriality, e.g., in response to increased sexual selection on females (Kraaijeveld et al. 2007), a subsequent transition to female territoriality could be facilitated by allowing females to co-opt sexually selected traits for functions in territorial competition. For example, male mate choice may select for large female body size and drive the loss of an ancestral male-biased size dimorphism. If body size is associated with resource-holding potential, size monomorphism subsequently provides a good vantage point for female territoriality. However, despite sexually monomorphic phenotypes, correlates of competitive success could differ between males and females (e.g., Koivula et al. 1993), such that sexual monomorphism would not directly facilitate female territoriality.

Independent of the sequence of transitions, a causal link between territoriality and sexual monomorphism predicts that male and female contestants should employ similar contest behavior and that contest outcome should depend on the same traits in males and females. We test this prediction in a sexually monomorphic cichlid fish of the genus *Tropheus*. Males and females actively defend separate feeding territories in the shallow littoral of Lake Tanganyika, East Africa, and browse on epilithic algae. The densely packed, contiguous territories range in size from 0.25 to 4 m^2^ (Takamura 1984; Sturmbauer et al. 2008), and observations of frequent replacements and territory expansions upon removal of territory holders (Yanagisawa and Nishida 1991) suggest strong intraspecific competition (Grant 1997). *Tropheus* are maternal mouthbrooders. Temporary pair-bonding occurs prior to spawning when females move into their mates’ territories for several days to weeks (Yanagisawa and Nishida 1991). Female mate preferences are influenced by male territory characteristics, but not by male size or color (Hermann et al. 2015). After spawning, females breed solitarily and establish new feeding territories once their fry are independent. Breeding takes place year-round, but long spawning intervals of females, probably required to recover from fasting during mouthbrooding, entail long periods of solitary territory defense (Yanagisawa and Sato 1990).

The genus *Tropheus* includes a small number of closely related, ecologically and morphologically similar species (Egger et al. 2007). Cichlid lineages basal to *Tropheus* (Salzburger et al. 2005; Koblmüller et al. 2008; Schwarzer et al. 2012) are sexually dimorphic in body size and color, with colorful territorial males and non-territorial, less conspicuously colored females. Sexual color and size dimorphism and male-only territoriality are also widespread in the “modern haplochromines” (Seehausen et al. 1998; Konings 2003; Genner and Turner 2005) to which the tribe Tropheini belongs phylogenetically (Salzburger et al. 2005). In contrast, females of most *Tropheus* species grow to the same size and display the same conspicuous, geographically variable color patterns as males (Konings 2013). Although currently available ecological and phylogenetic data have not allowed for fine-mapping of transitions in territoriality and sexual dimorphism in these species, the distribution of traits on a consensus tree suggests that *Tropheus* evolved from a sexually dimorphic ancestor with non-territorial females. Importantly, given polygynous mating (Sefc 2008; Steinwender et al. 2012), sexual dimorphism in *Tropheus* would be expected based on classic sexual selection theory. The key to the evolution of the male-like phenotypes of *Tropheus* females may therefore lie in social selection arising from competition for non-sexual resources (West-Eberhard 1983).

Large body size is generally associated with competitive success (Briffa and Sneddon 2007). However, the role of body size can be sex-specific, such that large body size is correlated with competitive success only in males, while female contests are determined by other factors such as perceived resource value (Robinson 1985; Koivula et al. 1993; Dale and Slagsvold 1995; Draud et al. 2004). In the present study, we test whether body size differences between contestants affect outcome differently in male and female contests. We also test whether males and females differ in the aggressive behavior shown during contests, as types of behavior differ in how much information on body size they provide (Keeley and Grant 1993). If female territoriality and sexual monomorphism are causally linked in *Tropheus* because body size plays an equally important role for the competitive success of male and females, we expect that both sexes show similar behavioral strategies in experimental contests and that the effects of body size asymmetries on the outcome are the same in both sexes. If, in contrast, males and females differ in these points, selection on body size arising from contest competition would also differ between males and females and provide no connection between territoriality and sexual size monomorphism.

In *Tropheus moorii*, the intensity of the conspicuous, sexually monomorphic body coloration could also influence competitive success. *T. moorii* is able to switch color patterns and intensities rapidly as means of intra- and interspecific communication. This makes the quantification of body coloration prohibitively difficult (Steinwender et al. 2012; Hermann et al. 2015) and is the reason why color is not included in the present analyses.

## Material and methods

### Animals

Wild-caught adult *T. moorii* of the population “Moliro” were imported by an ornamental fish trader. The Moliro population occurs in the southernmost part of DR Congo’s shoreline of Lake Tanganyika and is characterized by a dark red body color, a bright red dorsal fin, and a bright red stripe along the caudal peduncle. Fish were housed individually before the experiment (tank dimensions L × W × D, 60 × 35 × 30 cm). Both cultivation and experimental tanks were filtered with internal box filters, kept at 25–27 °C by an internal heater, and illuminated with an overhead white light on a 12:12 h light/dark cycle. Each fish was fed two portions of 150 mg food (alternating flakes and pellets) per day. The fish remained in the stock tanks of our laboratory after completion of the experiment.

### Experimental procedure

The experimental tank (L × W × D, 150 × 50 × 70 cm) was divided into a contest arena (46 × 46 × 35 cm) and an area furnished with 15 nursery cages (16.5 × 14 × 12.5 cm) in which the fish were kept before and during the series of trials in which they participated (Fig. S1). The nursery cages were wrapped with nontransparent sheets to prevent visual contact between their occupants and between the by-standing fish and the contest arena. The housing of test fish in the experimental tank avoided the physiological stress of frequent shifts between tanks. As, however, olfactory signals produced by contestants (Barata et al. 2007) could influence the behavior of fish in later contests, we confirmed that the number of previous contests on the same day had no effect on the activities of the contestants (generalized linear model testing the effects of contest number on the rates of charges and displays; all *p* values >0.2). Fish were measured (standard length, SL) before being placed in the experimental tank.

Using 13 females and 12 males, 46 female-female contests and 53 male-male contests were staged. Individual fish participated in 3 to 11 trials (mean *N* = 8), each time with a different opponent and a minimum interval of 72 h between contests. Pseudoreplication due to repeated use of individuals was controlled for in the statistical analysis. One to six contests took place per day between 09:00 and 15:00 hours. Male and female contests were staged in random order. A plastic tube (length 23 cm, diameter 10 cm) served as territory focus in the middle of the contest arena. For acclimation to the test arena, the two contestant fish were held in cylindrical wire cages, which were placed upright in the left and right side from the tube, for 20 min prior to being released simultaneously for the contest. During their confinement to the wire cages, the fish could see each other and the tube. The interactions of the contestants were videotaped for 15 min. Fish were watched throughout the trials, and four trials were terminated prematurely because of intense aggression. No injuries occurred. Subordinate fish were able to escape from attacks by retreating into a corner of the test arena, whereas the dominant individual remained near the tube.

Standard length (SL) (mean ± standard deviation) of females and males were 8.63 ± 0.42 and 7.82 ± 0.55 cm, respectively. Body size differences (mean ± standard deviation) between contestants were 0.48 ± 0.34 cm in female contests and 0.66 ± 0.48 cm in male contests. Body size asymmetry between contestants was represented by the relative size difference between contestants calculated as RSD = (focal fish SL − opponent fish SL)/mean SL of both. Mean and maximum absolute RSD were 0.06 and 0.14 in female contests and 0.08 and 0.21 in male contests.

### Data collection

The behavior of each of the two contestants was scored from the video playbacks with the help of the software Etholog 2.2.5 (Ottoni 2000). Charges (subject approaches opponent with a rapid movement) as well as lateral and frontal displays (subject displays to opponent laterally or frontally with erect fins and extended branchiostegal membrane) were tallied. Contests were considered resolved when one contestant (the “winner”) had gained sole ownership of the tube and did not tolerate its opponent near the tube. Resolution time was defined as the time between first interaction (first charge or display) and resolution of the contest. When contests were not settled within the 15-min trial and the contestants remained engaged in aggressive interactions, the contests were scored as ties. In these cases, contest duration was calculated as observation time minus time to first interaction. Escalations (mouth wrestling) were observed in 12 trials (eight male and four female contests; six settled and six tied contests) and were not included in the statistical analyses because of their infrequent occurrence. The data suggest no connection between escalation and body size differences between opponents, as RSD between mouth-wrestling contestants ranged from 0.01 to 0.18 (mean = 0.08).

### Statistical analyses

All analyses were conducted in R v. 3.1.0 (R Core Team 2013). A generalized linear model (GLM) with a binomial error distribution tested whether the likelihood of tied versus settled contests depended on sex and RSD between opponents and a likelihood ratio test (LRT) was used to compare nested models. A Cox regression model was used to test whether sex and RSD affected the probabilities of contest resolution through time (package “survival”; Therneau 2014). Contests finishing tied were accounted for by right-censoring the data. Additionally, linear models (LM) tested for effects of sex and RSD on the duration of contests that were resolved during the observation period.

Cumulative link models (CLM) were built in the package “ordinal” (Christensen 2010) to test for sex-specific effects of RSD on contest outcome, which was coded as an ordinal response variable (with levels “loser,” “tied,” and “winner”). As each contest yielded two correlated outcomes (winner /loser or tied/tied), we designated the contestants acclimating in the right wire cage as focus individuals and those acclimating in the left cage as their opponents. Results were cross-checked in an analysis assigning opposite roles to contestants. To control for the repeated use of individual fish, focus and opponent identities and the number of trials experienced by the focus and the opponent fish were included in the model. Fixed factors in the full model were sex, RSD, the interaction between sex and RSD, the number of trials experienced by the focus fish, and the number of trials experienced by the opponent fish; random factors were focus and opponent identities. Likelihood ratio tests (LRT) were used to compare nested models.

Differences in the rates of charges and frontal and lateral displays between male and female contests were tested by running generalized linear mixed models (GLMM) with negative binomial error distributions (“nbinom” or “nbinom1”, dependent on model AIC values) in the package glmmADMB (Skaug et al. 2013). A zero-inflation parameter was included when indicated by model comparisons based on AIC. The analyses considered activities that occurred before a contest was settled or, in case of tied contests, during the entire observation time and included contest duration as adjustment term (offset) in order to compare activities per time unit. Four different analyses addressed (1) activities per trial, i.e., sum of activities across the two contestants; (2) activities of contestants that won; (3) activities of contestants in tied contests; and (4) activities of contestants that lost. In (2), (3), and (4), focus and opponent identities were included as random factors to account for repeated use of individuals. In the analysis of tied contestants, correlations between contestants in the same contest were accounted for by including “contest” as a third random factor.

## Results

### Sex-independent effects of body size on contest outcome

The proportion of contests that was settled or remained tied during the 15-min observation time, was independent of body size asymmetry (absolute values of RSD) between contestants, and did not differ significantly between male and female contests (males: 72 % settled, i.e., 38 settled and 15 tied contests; females: 61 % settled, i.e., 28 settled and 18 tied contests; GLM: β_(sex)_ = 0.47 for males compared to females, *z* = 1.048, *p* = 0.30; β_(RSD)_ = 0.87 per unit RSD, *z* = 0.198, *p* = 0.84; no interaction between sex and RSD: LRT, *χ* ^2^ = 1.16, df = 1, *p* = 0.28).

**Fig. 1:**
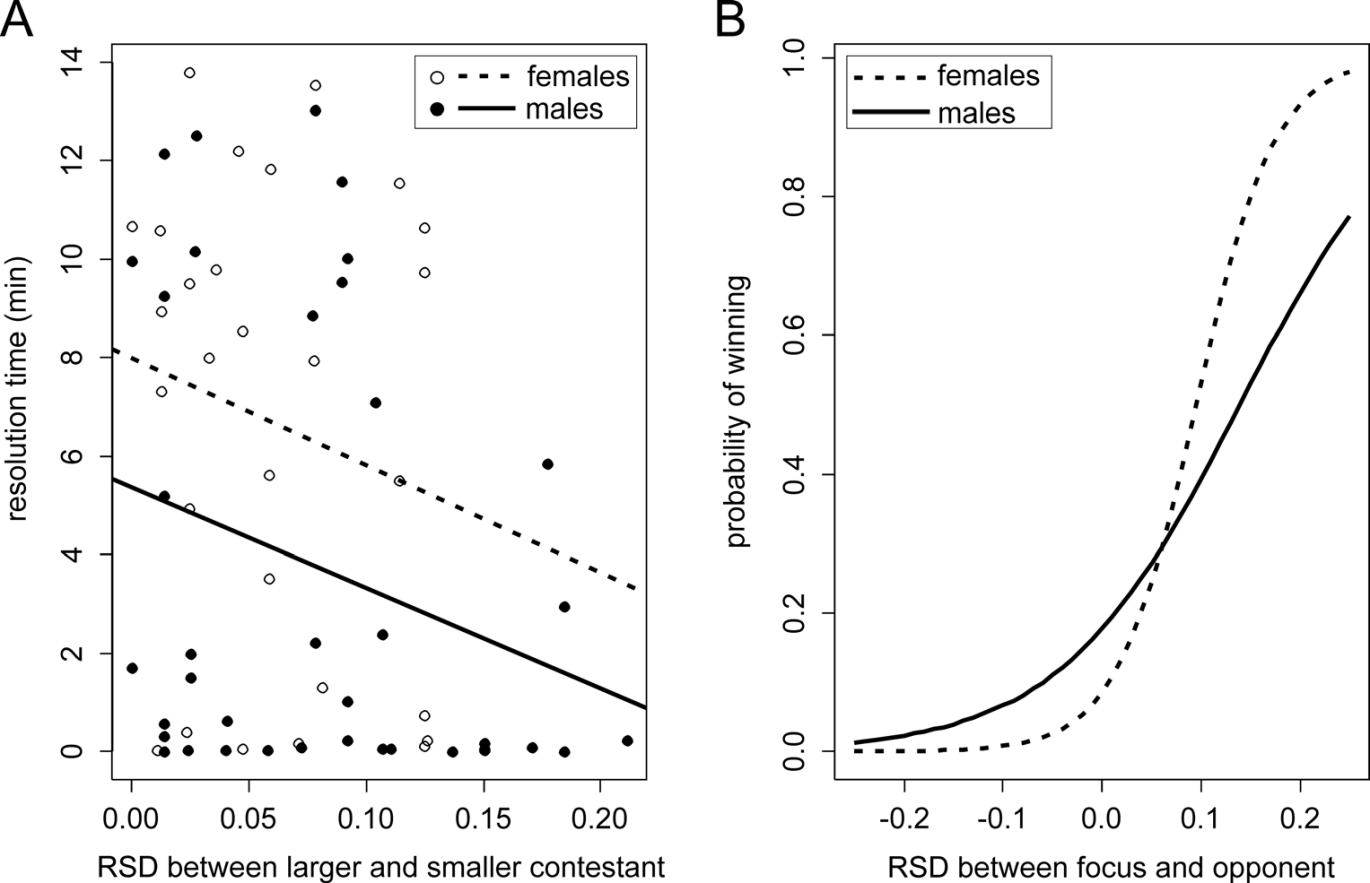
**a** Correlation between the resolution time of contests settled within the observation period and body size asymmetry (*RSD*, relative size difference) in male and female contests. **b** Relationship between an individual’s probability of winning a contest and its body size (dis)advantage relative to its opponent (RSD, relative size difference) predicted from cumulative link models fit to male and female contest data. The difference between male and female curves is not statistically significant (see text)

Neither sex nor body size had a significant effect on contest duration when both settled and tied contests were included in a Cox regression (β_(sex)_ = 0.71 for males compared to females, *z* = 1.54, *p* = 0.12; β_(RSD)_ = 5.83, *z* = 1.16, *p* = 0.25; interaction sex and RSD: LRT, *χ* ^2^ = 0.71, df = 1, *p* = 0.398). The lack of statistical effects in the Cox regression was probably due to the sex and size independency of ties. Restricting the analysis to those contests that were resolved within the 15-min observation time, both sex and RSD were correlated with resolution time (Fig. 1a). Females took longer to win (means ± sd: females, 6.7 ± 4.7 min; males, 3.7 ± 4.6 min; LM: β_(sex)_ = −2.544 min for males compared to females, *t* = −2.211, *p* = 0.031), and resolution time tended to increase with decreasing RSD (LM: β_(RSD)_ = −2.073 min per unit, *t* = −1.957, *p* = 0.055). The effect of RSD on resolution time was independent of sex (interaction sex and RSD: F_[1,62]_ = 0.004, *p* = 0.95).

We fitted cumulative link models to test for sex-specific effects of RSD on a contestant’s probability of losing, finishing tied, or winning. As the outcome of one contestant is dependent on the performance of its opponent, we arbitrarily selected the contestants acclimating in the right wire cage as focus individuals. The numbers of previous contests experienced by focus fish and by opponent fish had no effect on contest outcome and were removed from the model. There was no interaction between sex and RSD (LRT: *χ* ^2^ = 1.90, df = 1, *p* = 0.17) and no effect of sex (β_(sex)_ = −0.06 for males compared to females, *z* = −0.145, *p* = 0.89). The effect of RSD was significantly positive (β_(RSD)_ = 20.62, *z* = 3.66, *p* = 0.00025). Hence, the larger the size advantage of the focus fish over its opponent, the more likely the focus fish was to perform well in the contest, and the magnitude of the effect of RSD was the same in male and female contests. As expected, results for “left cage” contestants were nearly identical (not shown). The positive relationships between body size advantage and probability of winning predicted from models fit separately to male and female contest data are shown in Fig. 1b.

### Sexual differences in contest behavior

Male and female contests involved equal rates of charges, frontal displays, and lateral displays (activities of both contestants per minute contest duration; Table 1). Female and male winners employed equal rates of charges and displays. In tied contests, females showed higher display rates than males, and male losers charged at higher rates than female losers (Table 1). Contests settled within the observation time took longer in females, such that female winners performed a larger absolute number of charges and displays than male winners (GLMM: charges, β_(sex)_ = 0.78 for females compared to males, *z* = 2.09, *p* = 0.036; frontal displays, β_(sex)_ = 1.12 for females compared to males, *z* = 3.76, *p* = 0.0002; lateral displays, β_(sex)_ = 1.05 for females compared to males, *z* = 3.32, *p* = 0.0009).

**Table 1:** Sexual differences in charge and display rates during contests. Effect estimates β in the GLMM represent increased or decreased rates of activities in male contests compared to female contests

	Total (summed activities of both contestants)	Winners	Tied contestants	Losers
Charges	*β* = 0.298, *z* = 1.1, *p* = 0.27 (nbinom)	*β* = 0.098, *z* = 0.22, *p* = 0.82 (nbinom)	*β* = −0.385, *z* = −0.49, *p* = 0.63 (nbinom)	*β = 2.754, z = 3.49, p = 0.0005* (nbinom)
Frontal displays	*β* = −0.281, *z* = −1.42, *p* = 0.16 (nbinom)	*β* = −0.283, *z* = −1.06, *p* = 0.29 (nbinom)	*β = −0.993, z = −1.99, p = 0.05* (nbinom, ZI)	*β* = 0.419, *z* = 1.24, *p* = 0.21 (nbinom)
Lateral displays	*β* = 0.167, *z* = 0.64, *p* = 0.52 (nbinom)	*β* = −0.121, *z* = −0.55, *p* = 0.59 (nbinom)	*β = −0.644, z = −1.86, p = 0.06* (nbinom1)	*β* = 0.503, *z* = 1.42, *p* = 0.16 (nbinom, ZI)

Effects with *p* < 0.1 are highlighted in italics. *nbinom*, *nbinom1* dispersion parameter estimator used in glmmADMB, and *ZI* model included a zero-inflation parameter

## Discussion

### Male and female competition

A causal link between the evolution of sex-independent territoriality and the evolution of sexual monomorphism predicts that contest behavior and correlates of contest outcome are not sex-specific. In our experiment with the sexually monomorphic cichlid fish *T. moorii*, the effects of body size differences on contest duration and outcome did not differ between male and female contests. Given that males and females defend separate feeding territories, the competitive advantage of large individuals in the experiment suggests that contest competition selects for large body size in both sexes. Our finding stands in contrast to findings in hermit crabs in which males and females compete for the same resource, empty gastropod shells. In *Pagurus bernhardus*, males possess larger chelipeds than females, but cheliped size had no effect on the outcome of contests over shells (Briffa and Dallaway 2007), indicating that competition for shells does not oppose sexual dimorphism in this trait. In *Pagurus filholi*, the effects of body size on contest outcome were the same in males and females, and the existing male-biased size dimorphism was ascribed to strong sexual selection on male body size (Yoshino and Goshima 2002).

Males and females in our experiment showed the same types and similar rates of contest behavior. There were no sexual differences in charge and display rates scored across all contestants as well as in a separate examination restricted to winners of contests. Employing the same behavioral strategies, males and females apparently rely on the same body traits for succeeding in contest competition. Fish display body size and coloration in lateral displays by erecting dorsal and anal fins, spreading the caudal fin and extending the branchiostegal membrane. Frontal displays, with erect operculae and fins and extended branchiostegal membrane, likewise emphasize body size and display head coloration. Both displays will be most effective when performed by large and well-colored fish.

Sexual differences were detected in particular contexts (display rates in tied fights, charge rate by losers) as well as in resolution time and require specific experiments to test whether they originate, for instance, from sexual differentiation in assessment strategies or motivation (Draud et al. 2004). With longer time to contest resolution, female winners performed a higher absolute number of charges and displays than males. Contest behavior is energetically costly (Grantner and Taborsky 1998; Dijkstra et al. 2012), and average costs of female contests may therefore exceed those of male contests unless metabolic differentiation between the sexes allows females to perform contest behaviors more efficiently than males (compare with Dijkstra et al. 2012). With regard to the question addressed by the present study, it is important that those sex-specific patterns that were detected (i.e., females with higher display rates in tied contests and longer resolution time for settled contests) involve an increased display effort in females. This is consistent with the prediction that, in females like in males, competition for territories entails selection on traits supporting effective displays.

The present study does not test whether contestants perform differently in intra- and intersexual contests. Studies addressing sexual differentiation of contest characteristics often avoid intersexual contests (Draud et al. 2004; Arnott and Elwood 2009; Taves et al. 2009) because sexually motivated behavior can interfere with aggressive behavior (Cole et al. 1980). In preliminary trials, we attempted to circumvent this problem by testing females 8–21 days post-spawning when they would still be mouthbrooding under natural conditions. Days since spawning were the only (and positive) correlate of female contest performance (data not shown), indicating not only that the motivation or the physical condition to engage in contests was influenced by recent reproduction but also that recent spawners are unsuitable subjects to examine morphological correlates of contest performance.

Lacking experimental evidence, can we assume that patterns of male-female contests match those of intrasexual encounters? We are aware of one study in which correlates of contest outcome were compared between intra- and intersexual contests during the non-breeding season in a sexually monomorphic fish, *Gymnotus armorum* (Batista et al. 2012). No differences in the effects of body weight on contest outcome were detected between male-male, female-female, and intersexual contests. In a study of hermit crabs, components of contest behavior varied between males and females, but this variation was independent of whether the contest was intra- or intersexual (Briffa and Dallaway 2007). Contest behavior was also found to be independent of context in the sexually dimorphic convict cichlids, as gender-related differences in aggression observed during biparental brood care were maintained when non-breeding fish were tested solitarily (Arnott and Elwood 2009). These findings suggest that behavioral strategies can be retained in different contest situations. A possible proximate mechanism accounting for the consistent employment of particular behavioral tactics by vertebrate individuals involves patterns of cerebral lateralization, which can influence the effectiveness of different types of contest behavior (Arnott and Elwood 2009; Arnott et al. 2011).

### Loss of sexual dimorphism

The sex-independent effect of body size on competitive success is consistent with a role of territorial competition in the evolution of sexually monomorphic body size in the *Tropheus* lineage. However, the sequence of the transitions to female territoriality and monomorphism cannot be resolved with available data (Koblmüller et al. 2010), and other selection pressures may have driven the loss of sexual size dimorphism independent of female territoriality. We discuss possible scenarios in the light of our knowledge of the ecology and reproductive biology of *Tropheus*.

In the course of the radiation of the Tropheini from a generalist riverine ancestor into >20 endemic, ecologically diverse species in Lake Tanganyika (Salzburger et al. 2005; Koblmüller et al. 2010), *Tropheus* specialized on browsing filamentous algae covering the hard substrate in the shallow rocky littoral (Yamaoka 1997). According to the concept of economic resource defendability (Brown 1964), this specialization on an evenly distributed, predictable, and renewable food source may have promoted the defense of feeding territories by both sexes in *Tropheus*. In another taxonomic group, butterfly fish, plankton feeders usually forage in schools, whereas benthic feeders and particularly obligate corallivores are typically territorial (Roberts and Ormond 1992). Following the evolution of female territoriality in *Tropheus*, selection ensuing from territorial competition could then have driven the evolution of monomorphic body size.

Alternatively, increased sexual selection on females could have promoted the loss of sexual size dimorphism in *Tropheus* independent of the evolution of female territoriality (Kraaijeveld et al. 2007). However, the mating behavior of *Tropheus* entails considerably higher variance in the reproductive success of males than of females (Yanagisawa and Sato 1990; Yanagisawa and Nishida 1991; Sefc 2008; Steinwender et al. 2012), making strong sexual selection on females unlikely (Kraaijeveld et al. 2007). Predation pressure is another potential source of sex-independent selection for a particular body size. For example, shelter size constrains body size in mollusk shell-dwelling cichlids (Schütz and Taborsky 2005; Takahashi et al. 2009). In contrast, the cavities and crevices between layers of rocky substrate, into which *Tropheus* delve to avoid predation, provide shelter to cichlid species both considerably larger and smaller than *Tropheus* and are unlikely to enforce a narrow constraint on body size.

Female body size is positively correlated with clutch size in many fish taxa (Elgar 1990; Kolm et al. 2006), and fecundity selection could contribute to an increase in female body size (but see Shine 1988). *Tropheus* produce smaller clutches but larger eggs than related species. As egg size, size of young, and survival of young are positively correlated (Taborsky 2006), space requirements for the mouthbrooding of large eggs may have promoted an increase in female body size. Moreover, females browse algae to feed their young during buccal incubation, which entails agonistic interactions with conspecifics (Schürch and Taborsky 2005) that could also profit from large female body size.

Altogether, female body size appears to be more important in conspecific competition for food and territories than in connection with sexual selection and predation. A well-resolved phylogenetic reconstruction and comprehensive ecological data of related species may help to establish whether female territoriality indeed preceded the loss of sexual size dimorphism.

## Conclusion

Non-sexual social selection, i.e., differential reproductive success resulting from social competition for resources other than mates (West-Eberhard 1983), is a strong alternative to sexual selection in the endeavor to explain variation in levels of sexual dimorphism (LeBas 2006; Kraaijeveld et al. 2007; Tobias et al. 2012). Much attention has been paid to the role of female competition in the evolution of mutual ornamentation, including both competition for sexual resources such as access to mates and competition for other resources such as feeding territories and social status (Amundsen 2000; Rubenstein and Lovette 2009; Tobias et al. 2012). Like ornaments, body size functions both in mate choice and competition in many animals, and like mutual ornamentation, sexually monomorphic body size may be favored by intraspecific competition for resources. Our data support the hypothesis that selection pressure on body size due to intraspecific competition for territories does not differ between male and female *T. moorii*, such that female territoriality opposes the ancestral condition of male-biased size dimorphism. Importantly, while in other taxa, females often compete for resources related to reproduction (Clutton-Brock and Huchard 2013), female territoriality in *Tropheus* is not associated with access to mates, breeding opportunities, or brood care. In further agreement with predictions of social selection theory (West-Eberhard 1983), several *Tropheus* species also exhibit conspicuous, sexually monomorphic but geographically variable body coloration. Social selection arising from territorial competition could be a common mechanism underlying the evolution of sexually monomorphic body size and coloration in this lineage.

## Electronic supplementary material

Below is the link to the electronic supplementary material.

ESM 1 (GIF 87 kb)

High Resolution Image (TIFF 472 kb)
