# Supplementary figures and images for: Territorial competition and the evolutionary loss of sexual size dimorphism
Source: Behav Ecol Sociobiol. 2015 Jan 17;69(4):593–601. doi: 10.1007/s00265-014-1870-0 (PMC4359285; doi:10.1007/s00265-014-1870-0)

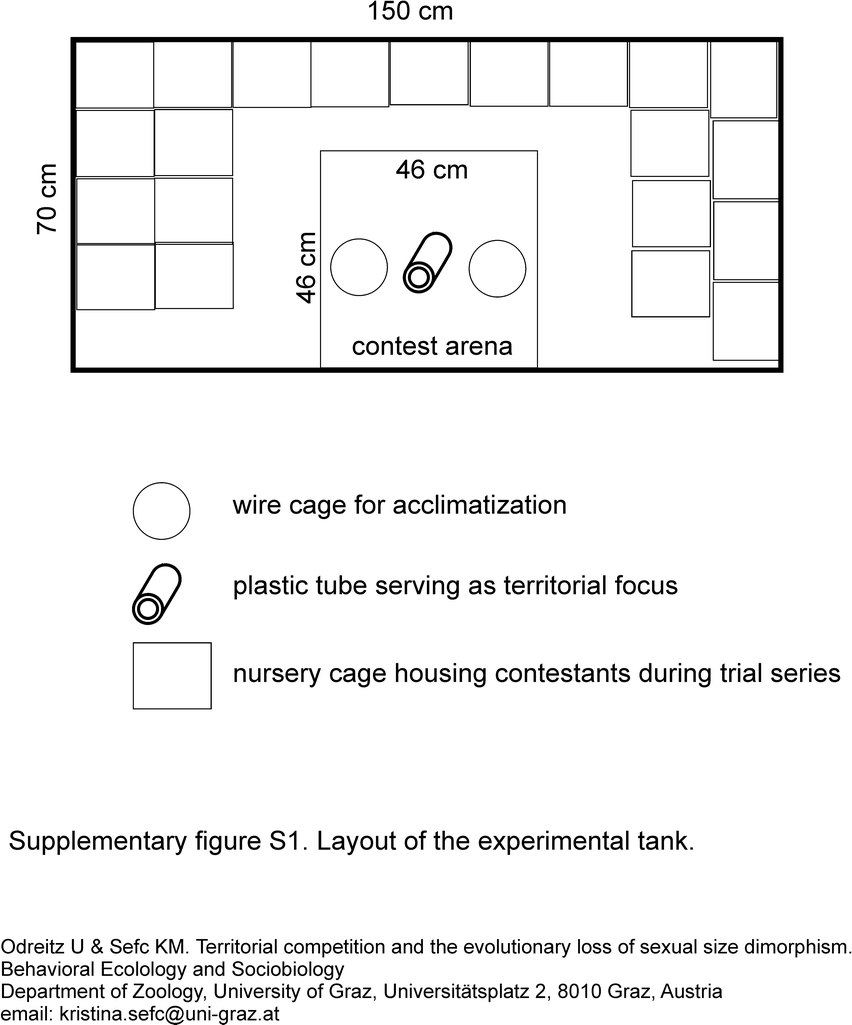

Supplement: Supplementary file 1 — (GIF 87 kb) [file 265_2014_1870_Fig2_ESM.gif]

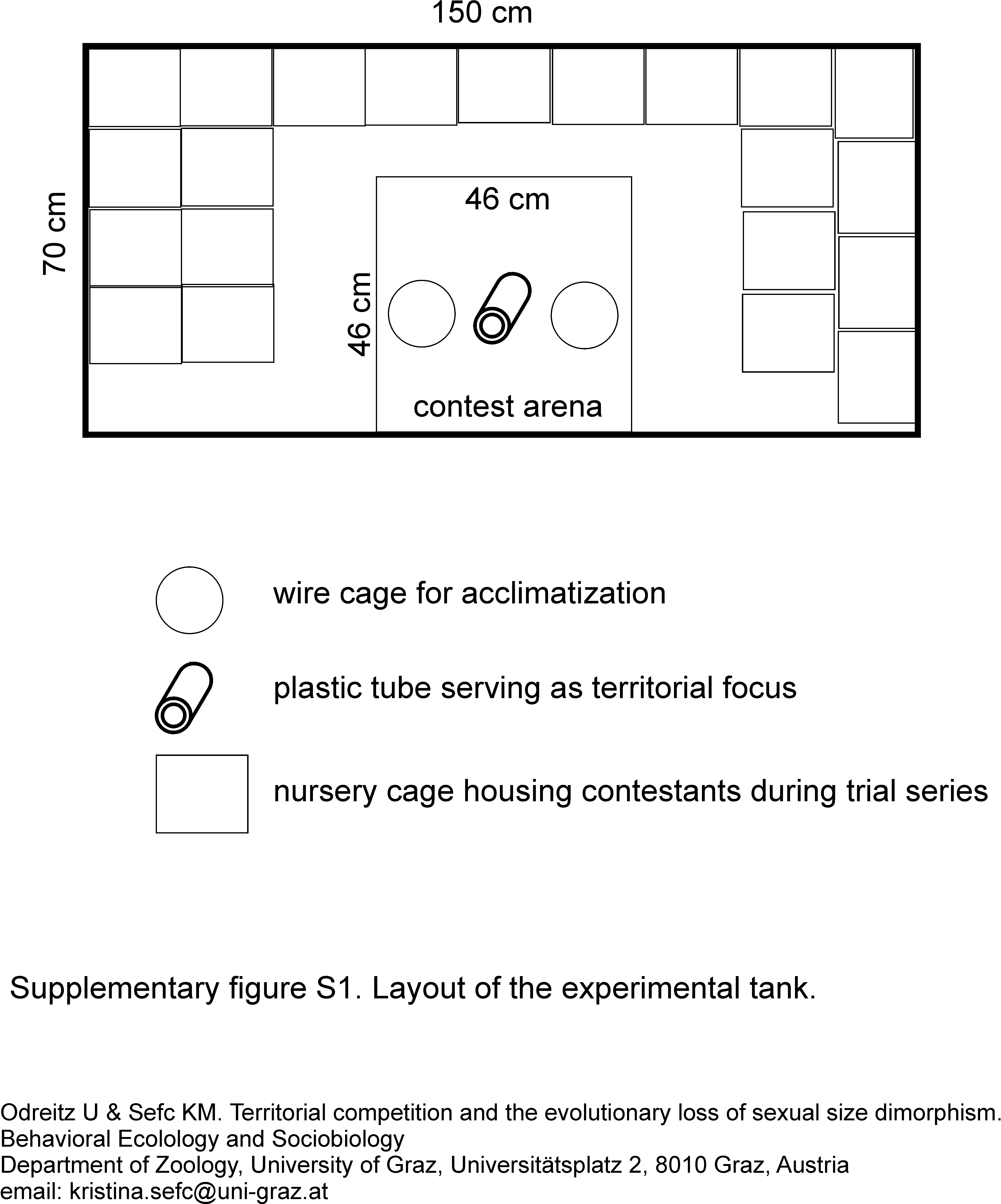

Supplement: Supplementary file 2 — High Resolution Image (TIFF 472 kb) [file 265_2014_1870_MOESM1_ESM.tif]
